# Supplementary material for: 3q26.2/MECOM Rearrangements by Pericentric Inv(3): Diagnostic Challenges and Clinicopathologic Features
Source: Cancers (Basel). 2023 Jan 11;15(2):458. doi: 10.3390/cancers15020458 (PMC9856433; doi:10.3390/cancers15020458)
Supplement: Supplementary file 1 [file cancers-15-00458-s001.zip › cancers-2119101-supplementary.pdf]

Supplemental Table S1:

Immunophenotypical features of cases with pericentric inv(3)/*MECOM-R* in this study

Antigen assessed	Total events* (n=17)	w -7/7q- (n=12)	w/o -7/7q- (n=5)
CD2	4/16	3/11	1/5
CD3	0/13	0/9	0/4
cytoCD3	0/13	0/9	0/4
CD4	9/17	6/12	3/5
CD5	1/17	1/12	0/5
CD7	12/17	10/12	2/5
CD10	2/3	1/2	1/1
CD13	17/17	12/12	5/5
CD14	0/17	12/12	0/5
CD15	0/17	0/12	0/5
CD19	2/17	2/12	0/5
CD22	1/11	1/7	0/3
CD25	6/13	4/9	2/4
CD33	17/17	12/12	5/5
CD34	17/17	12/12	5/5
CD36	2/7	1/5	1/2
CD38	17/17	12/12	5/5
CD41	0/11	0/8	0/3
CD45	17/17	12/12	5/5
CD52	2/3	1/2	1/1
CD54	3/5	2/3	1/2
CD56	6/17	4/12	2/5
CD64	3/17	2/12	1/5

CD71	7/7	5/5	2/2
CD117	17/17	12/12	5/5
CD123	17/17	12/12	5/5
CD133	1/17	12/12	5/5
HLA-DR	16/17	12/12	4/5
MPO	6/14	5/9	1/5
TdT	1/13	1/9	0/4

*Presented as ratio: number of positive cases / total numbers of cases assessed

w: with; w/o: without; -7: monosomy 7; 7q-: 7q deletion, including r(7)

Cases with -7 vs. cases without -7: no statistical significance ($p < 0.05$) was found for each marker assessed.

Cases with -7/7q- vs. cases without -7/7q-: no statistical significance ($p < 0.05$) was found for each marker assessed.

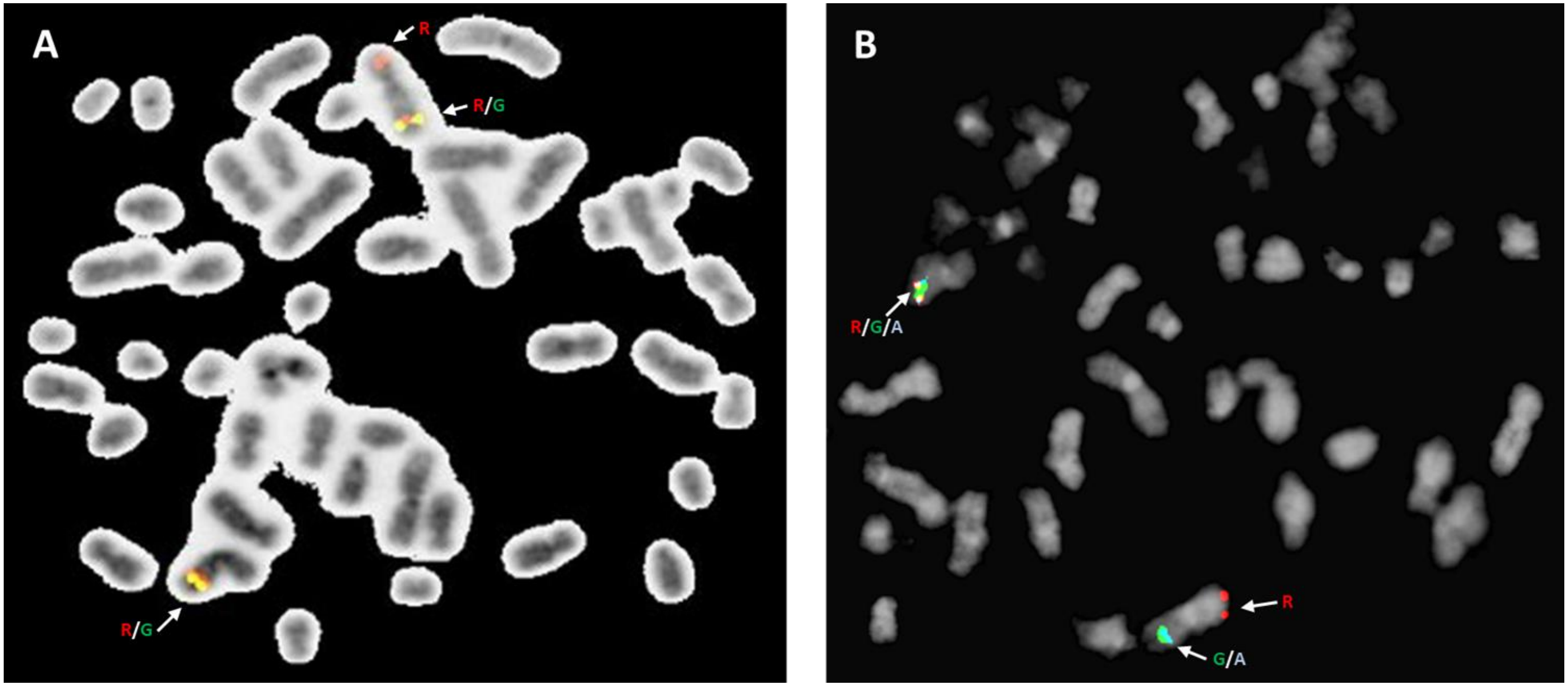

Supplemental Figure S1

MECOM FISH tests using two breakapart probe sets in Case #10. A. Commercial two-color breakapart probe; B. home-brew tri-color breakapart probe [51].
